# A comparative study between high-definition volumetric imaging computed tomography and multi-slice computed tomography in the detection of acute thoraco-lumbar disc extrusions in dogs

**DOI:** 10.4102/jsava.v92i0.2010

**Published:** 2021-03-11

**Authors:** Ross C. Elliott, Chad F. Berman, Remo G. Lobetti

**Affiliations:** 1 Department of Companion Animal and Clinical Studies, Onderstepoort, South Africa; 2 Bryanston Veterinary Hospital, Johannesburg, South Africa

**Keywords:** computed tomography, volumetric imaging, intervertebral disc disease, disc extrusion, multi-slice computed tomography

## Abstract

Computed tomography (CT) is commonly used to image intervertebral disc extrusion (IVDE) in dogs. The current gold standard for CT imaging is the use of multi-slice CT (MS CT) units. Smaller high-definition volumetric imaging (HDVI) mobile CT has been marketed for veterinary practice. This unit is described as an advanced flat panel. The goal of this manuscript was to evaluate the ability of the HDVI CT in detecting IVDE without the need for CT myelography, compared with the detection of acute disc extrusions with a MS CT without the need for MS CT myelogram. Retrospective blinded analyses of 219 dogs presented for thoraco-lumbar IVDE that had a HDVI CT (*n* = 123) or MS CT (*n* = 96) were performed at a single referral hospital. A total of 123 cases had HDVI CT scans with surgically confirmed IVDE. The IVDE was identified in 88/123 (72%) dogs on pre-contrast HDVI CT. The remaining 35/128 (28%) cases required a HDVI CT myelogram to identify the IVDE. Ninety-six cases had MS CT scans with surgically confirmed IVDE. The IVDE was identified in 78/96 (81%) dogs on the pre-contrast MS CT. The remaining 18/96 (19%) cases had a MS CT myelogram to identify the IVDE. Multi-slice CT detected IVDE significantly more than HDVI CT (*p* = 0.032). This study showed that the ability of HDVI CT for detecting IVDE is lower than that of MS CT. The HDVI CT system may be useful in smaller referral practices, with a lower case load where space is limited.

## Introduction

Thoraco-lumbar intervertebral disc disease is a common condition in the small animal patient (Griffin, Levine & Kerwin 2009). Decompressive surgery is the treatment of choice in treating thoraco-lumbar intervertebral disc disease in small animals. A hemilaminectomy is considered the technique of choice to decompress the spinal cord (Aikawa et al. 2012; McKee 1992; Langerhuus & Miles 2017).

Advanced imaging studies such as myelography, computed tomography (CT) or magnetic resonance imaging (MRI) are essential to correctly identify the site of disc extrusion (Cooper et al. 2014; Noyes et al. 2017; Robertson & Thrall 2011; Schroeder et al. 2011). Myelography is the most basic and most invasive imaging technique with the highest complication rate (Barone et al. 2002; Da Costa, Parent & Dobson 2011; Hecht et al. 2009; King et al. 2009). Magnetic resonance imaging has the highest rate of identification of thoraco-lumbar disc extrusion in dogs of around 98% (Cooper et al. 2014; Noyes et al. 2017; Robertson & Thrall 2011), but is relatively more expensive and time-consuming (25–145 min per examination) to acquire than both CT and myelography (4–45 min per examination) (Cooper et al. 2014; Hecht et al. 2009; Robertson & Thrall 2011).

Computed tomography is a rapid imaging modality that allows accurate detection of mineralised disc extrusions in dogs. Certain breeds have been shown to have a higher incidence of detection of intervertebral disc extrusions such as the dachshund (Hecht et al. 2009; King et al. 2009; Kranenburg et al. 2013; Newcomb et al. 2012; Schroeder et al. 2011). Detection rates of an acute disc extrusion using multi-slice (MS) CT are reported to be 88%, with markedly shorter scan times when compared with MRI (Cooper et al. 2014). A negative pre-contrast MS CT requires an additional MS CT myelogram or ideally an MRI, if there is a strong suspicion of a disc extrusion. A CT myelogram has similar complications to a radiographic myelogram (Hecht et al. 2009; Newcomb et al. 2012; Robertson & Thrall 2011).

Recently a high-definition volumetric imaging (HDVI) CT system was marketed for use in small animal veterinary practice; it was introduced to the South African veterinary industry in 2015 (Vimago, www.epicaanimalhealth.com). The unit is marketed as a mobile, smaller unit (230 centimetres [cm] × 150 cm footprint), lower operating costs, plug in and use to a single phase 220 volts (V) connection and lighter (600 kilograms [kg]) when compared with conventional MS CT units. The cost of the HDVI CT unit is similar to a conventional 16 slice CT unit, however, the set up, installation costs and infrastructure requirements are much less. The room housing the HDVI CT unit still requires radiation safety protocols as for MS CT.

High-definition volumetric imaging is a modification of an older cone beam CT (CBCT) technology. High definition imaging is defined as using advanced flat panel CT technology. It entails a cone shaped source of ionising radiation directed through the middle of the area of interest to an x-ray detector both of which are in a fixed position in the gantry (Scarfe & Farman 2008). During the rotation of the machine within the gantry, multiple (approximately 150 to more than 600) sequential planar projection images of the field of view are acquired in a complete, or partial, arc. High-definition volumetric imaging CT exposure incorporates the entire field of view, and only one rotational sequence of the gantry is necessary to acquire enough data for reconstruction (Scarfe & Farman 2008). These fields of view or stacks are available in two selectable options prior to scanning, a standard (265 millimetres [mm] × 215 mm) and a large (302 mm × 249 mm).

The spatial resolution is suggested to be equivalent to MS (4–16 slice) CT because of the decreased size of the voxels utilised. However, the speed of acquisition is markedly slower. Spatial resolution could be lost because of motion blur and negate the potential advantages (Scarfe & Farman 2008).

The capabilities of HDVI CT are unknown in small animal veterinary practice. However, the imaging ability of the HDVI CT unit needs to be determined as the cost of the unit itself is similar to a MS CT unit.

The aim of the study was to compare the ability of HDVI CT and MS CT imaging to detect acute thoraco-lumbar intervertebral disc extrusion in dogs. The ability of both HDVI CT and MS CT was determined by confirmation of the identification of site and side of disc extrusion at the time of decompressive surgery. It was hypothesised that the identification of acute disc extrusion in pre-contrast HDVI CT would be significantly lower in detection of intervertebral disc extrusion in the dog when compared with MS CT.

## Materials and methods

### Inclusion criteria

The records of Bryanston Veterinary Hospital were searched from 01 June 2017 to 01 June 2018 and from 01 November 2019 to 01 August 2020 for all files containing CT, disc extrusion, hemilaminectomy and spinal surgery. These records were re-examined to only include canine patients with a myelopathy affecting spinal cord segment T3-S1. To be included in the study, the dog had to have had a HDVI CT or a MS CT scan and an acute intervertebral disc extrusion with the surgical site and side of the dog confirmed after decompressive hemilaminectomy.

All dogs that had visible mineralised opacity in the vertebral canal or extradural compression on myelography consistent with an acute intervertebral disc extrusion were selected for the study. Exclusion criteria were: MRI scan as the initial imaging modality; other spinal cord pathology (mass, fibrocartilaginous embolism, meningomyelitis, non-compressive disc extrusion); no visible mineralised disc material on the pre contrast HDVI CT\MS CT and no extradural compression on the HDVI CT/MS CT myelogram; a chronic disc protrusion and presence of more than one disc extrusion at the time of presentation.

### Examinations

All dogs had a complete physical and neurological examination performed at the time of presentation. This isolated the pathology to the spinal segment T3-S1. A modified Frankel score was assigned to each patient to allow prognostication for the owner prior to proceeding with surgery (Levine et al. 2009). Patients older than 8 years of age had a full serum biochemistry and haematology performed. All patients in this study were determined to be clinically normal apart from the thoraco-lumbar disc disease.

### High-definition volumetric imaging computed tomography/multi-slice computed tomography technique

Dogs were premedicated with diazepam (valium 0.2 milligrams [mg]/kg IV, Roche, South Africa) and morphine (morphine sulphate 0.5 mg/kg IV, Pharma-Q, South Africa). Induction was performed with propofol (Diprivan 1% 6.6 mg/kg IV, AstraZeneca Pharmaceuticals, South Africa). Dogs were then intubated and maintained on isoflurane (Isofor, Safeline Pharmaceuticals, South Africa). The dogs were placed in dorsal recumbency to allow as little movement from breathing as possible to affect the quality of the spinal imaging. All dogs had fluoroscopic positioning to ensure optimal alignment of the vertebral column prior to commencement of the HDVI CT scan, centring over the thoraco-lumbar junction. This fluoroscopic positioning was performed in a ventro-dorsal plane and then in a latero-lateral plane to ensure the field of view included the vertebral column. The time taken from placement of the patient on the HDVI CT table until the patient was taken to surgical preparation was recorded as scan time.

**FIGURE 1:**
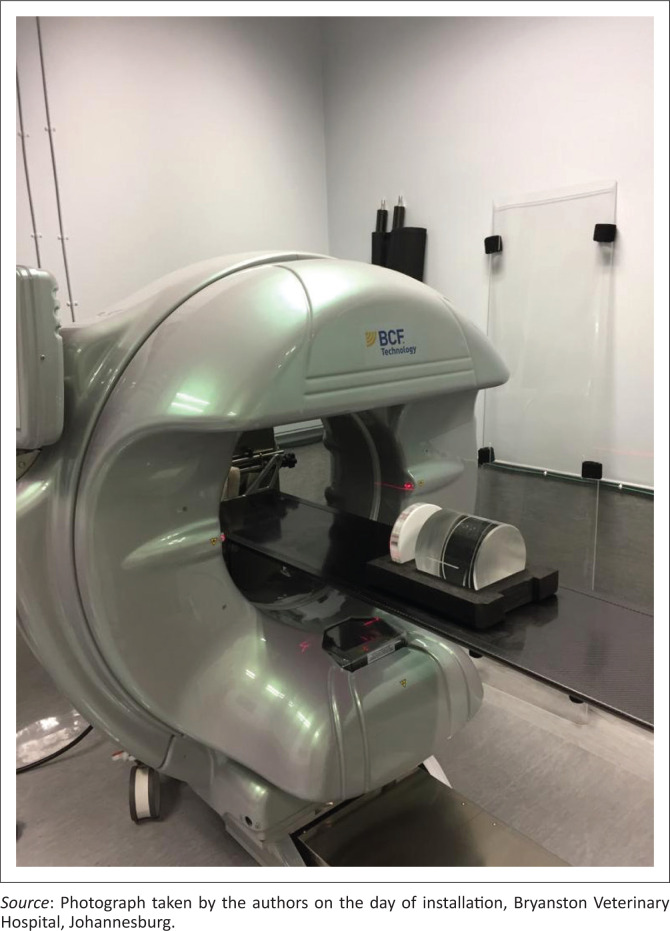
The high-definition volumetric computed tomography unit on wheels in a spare theatre converted to an imaging suite. The gantry rotates around the patient represented by the imaging phantom in this image.

All HDVI CT scans were performed using a high-definition volumetric CT system (Figure 1). All dogs that had a HDVI CT scan performed were subjected to a single or double volume acquisition or ‘stack’. Each volume acquisition took 30 s to perform a single rotation of the gantry. Images can be acquired at any angle and the gantry rotates a full 360-degrees. Images were reconstructed into axial slices of 300 micrometres (µm) and evaluated in 3-D reconstruction in all planes. The reconstruction took around 3 min.

In dogs that had a MS CT, all scans were performed using MS CT (GE Revolution ACT, General Electric Healthcare, USA). The time for the MS CT scans was recorded as stated here for HDVI CT scans.

**FIGURE 2:**
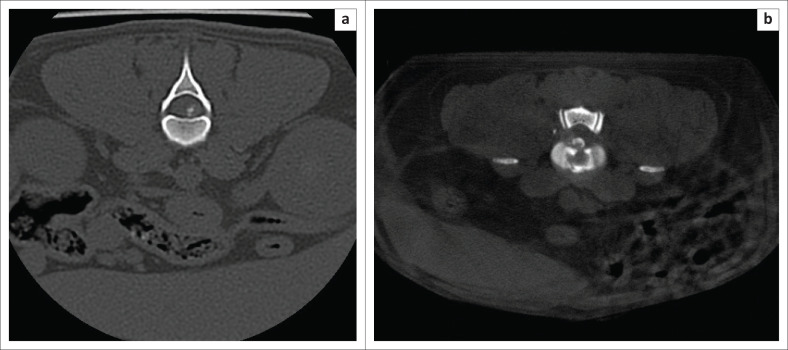
Comparative transverse images of an acute intervertebral disc extrusion at the 12th and 13th thoracic vertebrae in a (a) spine window (multi-slice computed tomography) (window width of 2000 and level of 500) and (b) bone window (high-definition volumetric computed tomography) (window width of 1500 and level of 500).

**FIGURE 3:**
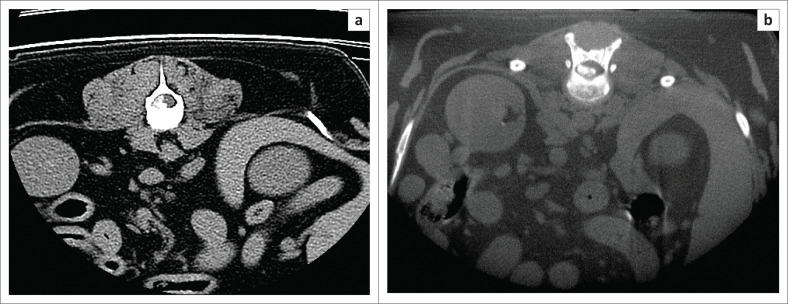
Comparative transverse images of an acute intervertebral disc extrusion at the 12th and 13th thoracic vertebrae between the soft tissue spine window (window width of 400 and level of 40) of a multi-slice computed tomography (a) and the high-definition volumetric computed tomography (b) soft tissue window (window width of 350 and level of 50).

**FIGURE 4:**
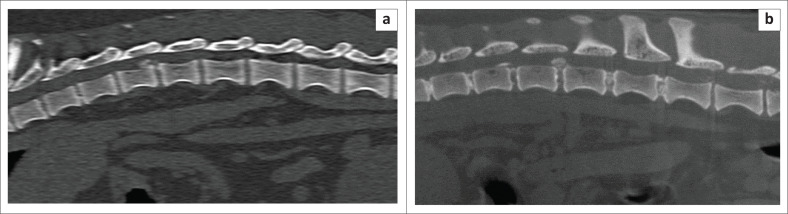
This figure shows comparative sagittal images of an acute disc extrusion at the 12th and 13th thoracic vertebrae on multi-slice computed tomography. Spine window (window width of 2000 and level of 500) (a) and high-definition volumetric computed tomography (b) in a bone window (window width of 1500 and level of 500).

The MS CT is a 16-slice CT unit whilst the HDVI CT is a fixed cone beam detector. A slice thickness of 1.25 mm with a 50% reconstruction increment to give a final slice thickness of 0.625 mm was used in the MS CT protocol. The slice thickness for the HDVI CT was 0.9 mm. Both the MS CT and the HDVI CT had a pitch of 1. The MS CT scans were performed with 120 kilovoltage peak (kVp) and 30 milliampere (mA). The HDVI CT scans were performed with a 90 kVp and a 3 mA. The image matrix for the MS CT was 512 by 512 and for the HDVI CT 536 by 536. The maximum field of view for the MS CT was 25 cm, an average of 10.6 cm was used on most dogs. The field of view of the HDVI CT is fixed in the standard setting of 18 cm × 18 cm. The 3D reconstruction algorithm for the MS CT was a spine (W2000 L500) and soft tissue spine (W400 L40). The 3D reconstruction algorithm for the HDVI CT was bone (W1500 L500) and soft tissue (W350 L50). All scans were performed in a transverse plane, all scans were reconstructed to sagittal, coronal and transverse planes (Figures 2–4).

In dogs with a negative pre-contrast HDVI CT/MS CT, a myelogram was performed. The dog was placed in ventral recumbency a small area over the L4-S1 area was shaved and aseptically prepared. A 22 gauge spinal needle was placed into the sub-arachnoid space at L5-6. Placement was confirmed with injection of 0.3 millilitres (mL) of Iohexol (Omnipaque, 0.3 mL/kg, 140 mg/mL, GE Healthcare, USA); a fluoroscopic image was taken to confirm placement of the needle and the contrast. A further 2 mL of Iohexol was injected and a thoraco-lumbar HDVI CT myelogram was performed as for the pre-contrast scan. The time taken to perform the myelogram was included in the record of the HCVI and MS CT myelography time, which was added to the pre-contrast scan times to give a total scan time.

### Surgical procedure

After the HDVI CT or HDVI CT myelogram, all dogs were clipped and aseptically prepared for surgery. All dogs had a standard described hemilaminectomy to provide access to the vertebral canal for decompression of the spinal cord (McKee 1992). The site of disc extrusion was confirmed by direct visualisation at the time of surgery.

### Data extraction

The HDVI CT and MS CT images linked to the patients file were analysed by a specialist surgeon and an internal medicine resident (R.C.E. and C.F.B.) blinded to the name of the patient, the need for further imaging (HDVI CT/MS CT myelogram or MRI), surgical history or outcome of the decompressive surgery. Image interpretation was performed using Easy Image with images viewed in a spine window (W2000 L500) and soft tissue spine (W400 L40) for MS CT, and a bone window (W1500, L500) and a soft tissue window (W350, L50) for HDVI CT. Multiplanar reformatting and volume rendered techniques facilitated interpretation. All CT scans were examined by the authors (R.C.E. and C.F.B.) prior to surgery.

The determined disc space and side of disc extrusion were recorded on the HDVI CT and the MS CT. If this was not possible it was recorded as a negative detection and the file was checked for a HDVI CT/MS CT myelogram. These data were then checked with the surgical report on file for that patient. The breed of dog, age of dog, disc space affected and side of disc extrusion were recorded.

The dogs were placed into two groups: group 1 dogs had an HDVI CT and group 2 dogs had a MS CT performed. Each of these two groups were then further spilt into dogs where the acute disc extrusion was detected on pre-contrast imaging and dogs where the acute disc extrusion required a myelogram to detect the site of acute disc extrusion (Table 1).

**TABLE 1:** Detection of acute intervertebral disc extrusions in different breeds of dogs using high-definition volumetric computed tomography, high-definition volumetric computed tomography with myelography, multi-slice computed tomography and multi-slice computed tomography with myelography.

Breed	Acute disc HDVI CT	Acute disc HDVI CT M	Acute disc MS CT	Acute disc MS CT M	Total
Dachshund	64	14	53	6	137
Pekingese	12	9	15	9	45
Other Breeds	12	12	10	3	37
**Total**	**88**	**35**	**78**	**18**	**219**

HDVI CT, high-definition volumetric computed tomography; MS CT, multi-slice computed tomography; M, myelography.

### Statistical analysis

Using Prism 8 (GraphPad Software, USA), correlations between positive and negative diagnosis of acute IVDD using MS CT versus HDVI CT compared with surgical findings were determined by using Mann–Whitney and Wilcoxon test. Correlations between the time taken for a MS CT, MS CT myelogram, HDVI CT and a HDVI CT myelogram were determined by using Mann–Whitney and Wilcoxon test. Values of *p* < 0.05 were considered significant.

### Ethical considerations

Ethical clearance was not needed for the study. All work was carried out for clinical cases treated with the current standard of care recommended in veterinary medicine. All data were gathered from clinical cases treated at the Bryanston Veterinary Hospital.

## Results

A wide range of breeds was presented in this study for suspected thoraco-lumbar intervertebral disc extrusion with the two most common breeds being the dachshund followed by the pekingese (Table 1).

In total, 123 dogs had HDVI CT scans performed. The intervertebral disc material was identified on pre-contrast HDVI CT in 88/123 (72%) dogs. All the remaining 35/123 (28%) dogs had a HDVI CT myelogram.

In total, 96 dogs had MS CT scans. The intervertebral disc material was identified in the pre-contrast MS CT in 78/96 (81%) dogs. All of the remaining 18/96 (19%) dogs had a MS CT myelogram performed.

When comparing the MS CT with the HDVI CT across all dogs in the study, the MS CT detected acute disc material in a significantly higher number of dogs than HDVI CT (*p* = 0.032).There was, however, no significant difference in the ability of HDVI CT versus MS CT in the identification of acute intervertebral disc extrusion in dachshunds and pekingese. In the dachshund, HDVI CT detected 64/78 (82%) of acute intervertebral disc extrusions and the MS CT detected 53/59 (89%) of acute intervertebral disc extrusions. In the pekingese, HDVI CT detected 12/21 (57%) acute intervertebral disc extrusions and MS CT detected 15/24 (63%) acute intervertebral disc extrusions. However, in another breed of dogs, there was a statistically significant difference (*p* < 0.012) between HDVI CT and MS CT where HDVI detected 12/24 (50%) of acute intervertebral disc extrusions and the MS CT detected 10/13 (77%) of acute intervertebral disc extrusions.The mean scan time for HDVI and MS CT were 12 min (range 8–17) and 5 min (range 4–12), respectively, with HDVI CT having a significantly increased scan time on average when compared with MS CT (*p* = 0.035). The mean scan times for HDVI CT myelogram and MS CT myelography were 36 (range 22–55) and 31 min (range 14–56), respectively, with no significant difference between the myelography scan times.

## Discussion

High-definition volumetric imaging CT has recently been marketed to the veterinary field. The manufacturers state that it is comparable with MS CT technology. Our aim was to determine the number of acute intervertebral disc extrusions in dogs detected with HDVI CT confirmed by decompressive surgery. This was then compared with the number of acute disc extrusions in dogs detected by a newer MS CT unit at a single referral hospital. Given the fact HDVI CT has very little dedicated literature supporting its use in small animal practice, the ideal uses and limitations of HDVI CT are not known.

In this manuscript, the hypothesis was supported in the sense that across all breeds of dogs, pre-contrast HDVI CT detected acute intervertebral disc extrusions less often when compared with pre-contrast MS CT. Whilst the detection of acute intervertebral disc extrusion with pre-contrast HDVI CT was less than that of MS CT, it was still higher than the detection of acute intervertebral disc extrusion reported for plain film radiographs (Kirberger, Roos & Lubbe 1992).

In the dachshunds and pekingese, however, there was no significant difference between the detection of acute intervertebral disc extrusion with pre-contrast HDVI CT and MS CT. Given that these are the most common breeds presented for acute disc extrusion, the unit may be useful in a smaller referral practice with a lower case load looking to increase imaging capabilities without the infrastructure upgrades required for a MS CT unit. However, HDVI CT detected acute intervertebral disc extrusion in other breeds of dogs in a lower percentage of cases than MS CT, thus limiting the versatility of the HDVI CT unit in such cases. The potential need for an invasive HDVI CT myelogram in a high number of dogs in this demographic of patients’ needs to be weighed up against the potential benefits of mobility and size of the HDVI CT unit versus a MS CT unit.

Both CT modalities show a markedly shorter scanning time than any MRI unit reported in the literature (Robertson & Thrall 2011). In addition, MS CT pre-contrast scans were significantly shorter than HDVI CT scan times. This was because of the increased need for measuring and positioning of the dog and the increased time for stacked image acquisition with HDVI CT compared with the helical acquisition of MS CT. However, there was no significant difference in time between HDVI CT myelography and MS CT myelography. This was because of the fact that the myelogram process for HDVI CT and MS CT was similar in regard to needle placement and contrast injection. The effects of anaesthetic time on outcomes remains controversial with smaller studies saying that there is minimal effect on outcome whilst a recent study shows increased morbidity with an increased anaesthesia time (Bos et al. 2012; Bottcher et al. 2013; Davis & Brown 2002; Fenn et al. ; Moore, Early & Hettlich 2016).

The increased scan time of the HDVI CT is significantly longer than the MS CT unit and hence can be affected more by motion blur during image acquisition, necessitating scans to be done in dorsal recumbency to limit the effect of motion or respiration on the vertebral column.

## Conclusion

This study showed that the pre-contrast HDVI CT allows identification of the correct disc space and side of an acute intervertebral disc extrusion in 72% of the dogs without the need for HDVI CT myelography. This is significantly lower than the pre-contrast MS CT detection of acute intervertebral disc extrusion in dogs seen in this study of 81% without the need for MS CT myelography. This study supported the use of HDVI CT unit in smaller referral practices if the caseload is higher in chondrodystrophic breeds, given that the detection of pre-contrast HDVI CT was similar to pre-contrast MS CT for chondrodystrophic breeds.

### Limitations of this study

One of the major limitations of this study was that a specialist surgeon and not a diagnostic imaging specialist performed the interpretation of the HDVI CT and MS CT scans. With the technology being new to the surgeon in the beginning there may have been a lack of experience/confidence in the modality, which may have decreased the ability to identify the correct disc space and side. This may have led to an increased frequency of performing a CT myelogram. Hopefully, the large number of cases comparable to other similar studies would negate this effect.

A second limitation was that the majority of our study population were dachshunds given the prevalence of acute intervertebral disc extrusions in this breed and the popularity of the breed in South Africa. This may have skewed the results of the study.

The final limitation is that no two dogs had both a HDVI CT and a MS CT performed to directly compare the modalities. The hope is that the high case numbers would minimise this effect.
